# DyMamba: dynamic Mamba for microscopy image semantic segmentation

**DOI:** 10.1093/bioinformatics/btag391

**Published:** 2026-06-17

**Authors:** Buqing Cai, Xingsheng Wang, Zhuo Jia, Fa Zhang, Bin Hu, Xiaohua Wan

**Affiliations:** Key Laboratory of Brain Health Intelligent Evaluation and Intervention, Ministry of Education, Beijing Institute of Technology, Beijing, 100081, China; School of Medical Technology, Beijing Institute of Technology, Beijing, 100081, China; Key Laboratory of Brain Health Intelligent Evaluation and Intervention, Ministry of Education, Beijing Institute of Technology, Beijing, 100081, China; School of Medical Technology, Beijing Institute of Technology, Beijing, 100081, China; Key Laboratory of Brain Health Intelligent Evaluation and Intervention, Ministry of Education, Beijing Institute of Technology, Beijing, 100081, China; School of Medical Technology, Beijing Institute of Technology, Beijing, 100081, China; Key Laboratory of Brain Health Intelligent Evaluation and Intervention, Ministry of Education, Beijing Institute of Technology, Beijing, 100081, China; School of Medical Technology, Beijing Institute of Technology, Beijing, 100081, China; Key Laboratory of Brain Health Intelligent Evaluation and Intervention, Ministry of Education, Beijing Institute of Technology, Beijing, 100081, China; School of Medical Technology, Beijing Institute of Technology, Beijing, 100081, China; Key Laboratory of Brain Health Intelligent Evaluation and Intervention, Ministry of Education, Beijing Institute of Technology, Beijing, 100081, China; School of Medical Technology, Beijing Institute of Technology, Beijing, 100081, China

## Abstract

**Motivation:**

Segmentation of cell bodies and organelles in microscopy images is critical for biological research, particularly in scenarios with multiple regions of interest where spatial continuity is essential. The Mamba architecture, derived from State Space Models (SSMs), has recently gained attention for efficiently modeling long-range dependencies in sequences, achieving excellent results in both natural and medical image segmentation. However, in vision tasks, current Mamba scanning strategies mainly focus on raster-scanning and local-scanning, which introduce spatial discontinuities, severely affecting the effectiveness of segmentation at the pixel level, especially in dense segmentation tasks.

**Results:**

In this article, we propose DyMamba, a Mamba-based model featuring a dynamic scanning strategy that adaptively plans scanning paths based on local features and complexity. In addition, to address the challenges of detail prediction and small object detection, we introduce a local aware module that performs pixel-level regional processing on images. DyMamba achieves robust segmentation across diverse microscopy image types, including cell-, organelle- and tissue-scale images. Experiments on six datasets and multiple scanning strategies demonstrate the excellent performance of our method in segmenting microscopy images, achieving an average improvement of 6.9% in mDice and 4.3% in mIoU over state-of-the-art methods across all datasets.

**Availability:**

The code is released at https://github.com/cbqBit/dymamba.

## 1. Introduction

Microscopy image segmentation plays a key role in biology and medicine, and can be used to segment cells, tissues, and lesion regions (Meijering 2012, Schindelin *et al*. 2012, Boutros *et al*. 2015, Cutler *et al*. 2022). High-precision segmentation helps to accurately count cells and organelles, and also aids in structure localization and lesion detection in the clinic (Deng *et al*. 2020, Greenwald *et al*. 2022, Wang *et al.* 2024). With the development of deep learning, research in the field of microscopy image has made great progress, especially in automatic microscopy image segmentation (Caicedo *et al*. 2017, 2019, Ma *et al*. 2024b, Archit *et al*. 2025). CNN-based and Transformer-based networks, for instance, U-net, UNETR and Swin-UNETR, has been widely used in microscopy image segmentation (Ronneberger *et al*. 2015, Hatamizadeh *et al*. 2022, 2021, Stringer and Pachitariu 2025).

However, precise microscopy image segmentation is a challenge, given its need to integrate local features with global contextual information, particularly in segmentation tasks across different microscopy modalities (Liu *et al*. 2024b, Dong *et al*. 2025, Xiao *et al*. 2025, Zhu *et al*. 2025). Microscopy images are characterized by high resolution, low contrast, noise interference, complex imaging conditions, varying target sizes, and a large number of dense regions of interest. Although CNN-based and Transformer-based models are two dominant methods, they still have limitations in handling long sequences and high-resolution tasks respectively (Shi *et al*. 2024, Zhang *et al*. 2024). CNN-based methods are limited by the local receptive fields, which have insufficient ability to deal with long-range information and insufficient feature extraction (Xu *et al*. 2024, Liu *et al*. 2024a). In addition, although the transformer-based method has global modeling capability, it requires a large amount of computational resources, which degrades the efficiency of segmentation task across different modalities.

Recently, Mamba, which originates from State Space Models(SSMs), has shown its efficiency and effectiveness in long-range dependency modeling, maintaining these capabilities while scaling in a near-linear way as the sequence length increases (Gu and Dao 2024, Liu *et al*. 2024b). Recent studies, such as VMamba, Vision-Mamba, U-Mamba, and Swin-UMamba, etc., have shown that mamba also achieves good results in vision tasks (Zhu *et al*. 2024, Liu *et al*. 2024a, Ma *et al*. 2024a). Mamba-based medical segmentation has advanced further, featuring Mamba-Sea (Cheng *et al*. 2025) for domain generalization via global-to-local augmentation, SegMamba-V2 (Xing *et al*. 2026) as a 2M-parameter 3D framework surpassing nnU-Net, and HybridMamba (Wu *et al*. 2025) for boundary-sensitive dense segmentation with a dual-domain architecture. The scanning strategy is crucial for visual mamba, as it determines the order in which 2D visual data is converted into a 1D sequence (Liu *et al*. 2024b). Different scanning methods have different effects in visual mamba, and existing methods can be mainly categorized into the following types. BiDirectional Scan and Cross-Scan are the most widely used multidirectional sequential scanning strategies, which adopt 1D bidirectional scanning and 2D four-directional scanning, respectivelyvision-mamba, vmamba. Additionally, some methods also incorporate local scanning and skip scanning (Huang *et al*. 2024, Pei *et al*. 2025). However, these scanning methods are limited in high-resolution, target-dense microscopy images segmentation tasks due to their predefined paths, inability to dynamically adjust to inputs, and spatial discontinuity issues.

Given the characteristics of microscopy images featuring multiple regions of interest and targets, the introduced spatial discontinuities can affect feature extraction performance. The existing Mamba model introduces spatial discontinuities due to its predefined scanning path (Xiao *et al*. 2025), and its feature transformation heavily relies on sequence relationships, further limiting the effectiveness of sequence modeling. In this work, we introduce DyMamba, a Mamba-based framework that combines a novel dynamic scanning strategy for 2D microscopy image segmentation. Different from previous Mamba-based networks, DyMamba uses a dynamic scanning strategy in Mamba blocks. This approach aims to resolve spatial discontinuities caused by prior scanning strategies, thereby enhancing performance in multimodal microscopy image segmentation tasks.

To sum up, our contributions can be summarized as follows:

- We introduce a *dynamic scanning* strategy to the SSM block, namely the DS-VSS block, which contains a dynamic scanning SS2D(DS-SS2D) block, dynamically sets the paths based on the feature points. This DS-VSS block can effectively resolve spatial discontinuities and improve the accuracy of boundary segmentation in tasks with multiple regions of interest.
- We propose a *local aware* module that performs feature extraction on images at the pixel level. This module first divides the input image into patches, performs local spatial modeling, and obtains local feature dependencies, aiming to improve the segmentation effect of small objects.
- We provide a comprehensive method, DyMamba, for various types of microscopy image segmentation. This method effectively alleviates the spatial discontinuity caused by the scanning strategy of Mamba in visual tasks, achieving good results on six datasets under various imaging conditions and multiple modalities.

## 2. Method

### 2.1. Overview

**Figure 1:**
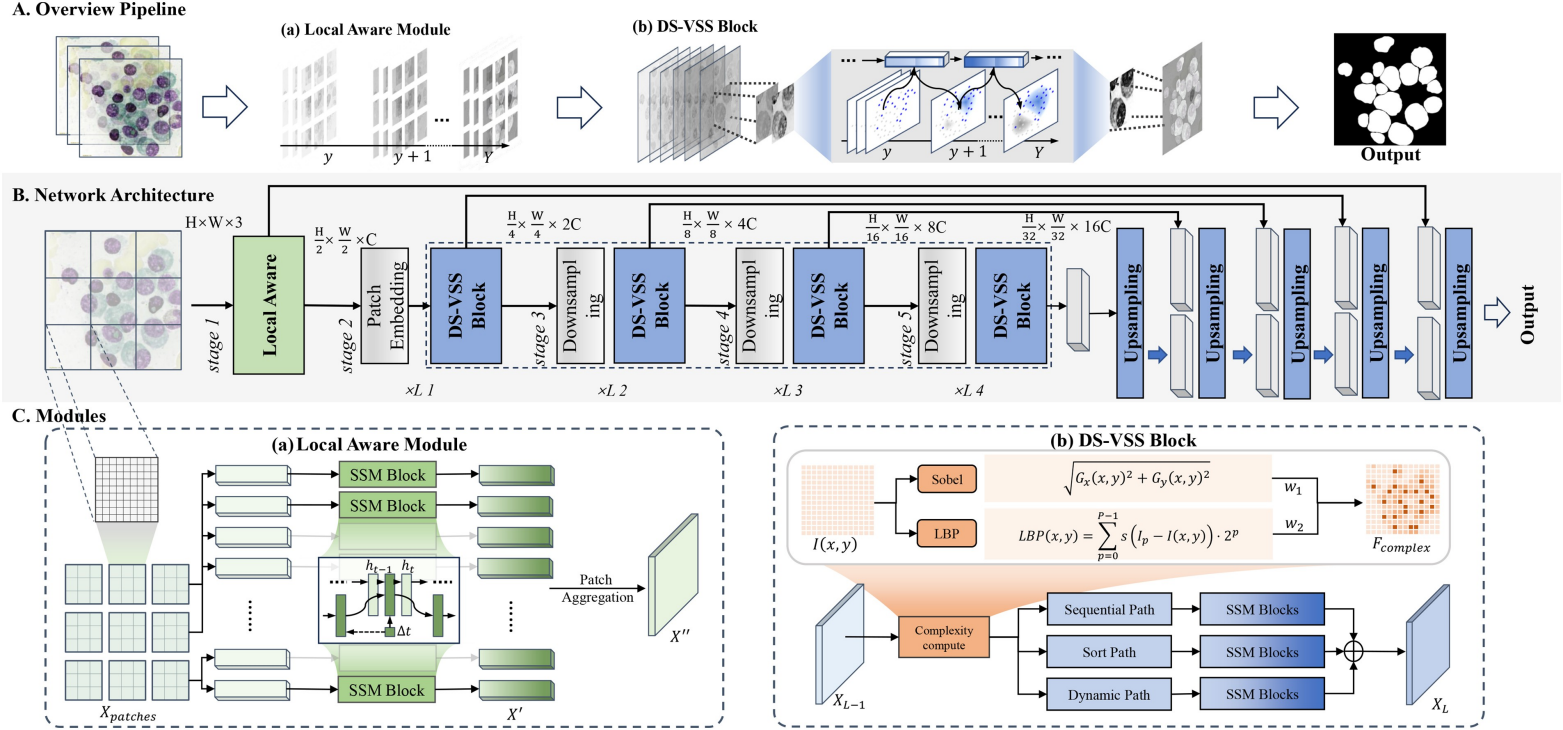
Network architecture overview. (A) Overview of the pipeline. Input images sequentially pass through the local aware module and the Dynamic Scanning-Visual State Space (DS-VSS) blocks to generate segmentation masks. (B) Network structure. An encoder-decoder design featuring a four-stage encoder and a five-stage decoder with upsampling. (C) Module details. (a) Local aware module partitions images into patches for SSM-based feature extraction with spatiotemporal fusion; (b) DS-VSS block computes complexity map and outputs feature maps.

The overall architecture of DyMamba is illustrated in Fig. 1. As is shown in Fig. 1A, it mainly consists of: (a) a local aware module and (b) DS-VSS Blocks. Fig. 1A depicts the overview pipeline of segmentation tasks. Images sequentially pass through the local aware module and the DS-VSS blocks to extract features, ultimately obtaining the segmentation results.

As is shown in Fig. 1B, given a 2D image $I\in R^{H\times W\times 3}$, where *H* and *W* refer to the height and width of the input, respectively. Firstly, it goes through the local aware module to get the processed module $X_{\mathrm{local}}$ with a shape of H×W×C. Next, this part is passed as input through the dynamic scanning module to get four layers of feature maps of different sizes $F_{e}\in R(H{/2}^{i})\times (W{/2}^{i})\times (C\times 2^{i-1})$, where i∈{1,2,3,4,5}, and *C* is obtained by the Patch Embedding layer. Finally, the obtained feature layers are fed into the head network for segmentation.

### 2.2. Preliminaries: SSM models

SSM Models can be formulated as the following Ordinary Differential Equations:


(1) $$\begin{array}{c} h^{\prime }(t)=Ah(t)+Bx(t)\\ y(t)=Ch(t) \end{array}$$

Where matrix $A\in R^{N\times N}$ contains the evolution parameters; $B\in R^{N\times 1}$ and $C\in R^{1\times N}$ are projection matrices.

However, the continuous-time equation in Equation (1) are difficult to solve using deep learning. In practice, this equation is usually transformed into a discretized form via the zero-order hold rule, and the discretized Ordinary Differential Equations are given by:


(2) $$\begin{array}{cc} & h(t)=\bar{A}h(t-1)+\bar{B}x(t),\\ & y(t)=Ch(t). \end{array}$$

Where $\bar{A}$ and $\bar{B}$ are the discrete version of A and B. For efficient implementation, the iterative computations in Equation (2) can be performed in parallel with a global convolution operation:


(3) $$\begin{array}{c} y=x⊛\bar{K}\\ \text{with}\;\bar{K}=(C\bar{B},C\bar{AB},\ldots ,C{\bar{A}}^{L-1}\bar{B}), \end{array}$$

Where ⊛ denotes the convolution operator and $\bar{K}\in R^{L}$ the SSM kernel.

### 2.3. Dynamic scan module

**Figure 2:**
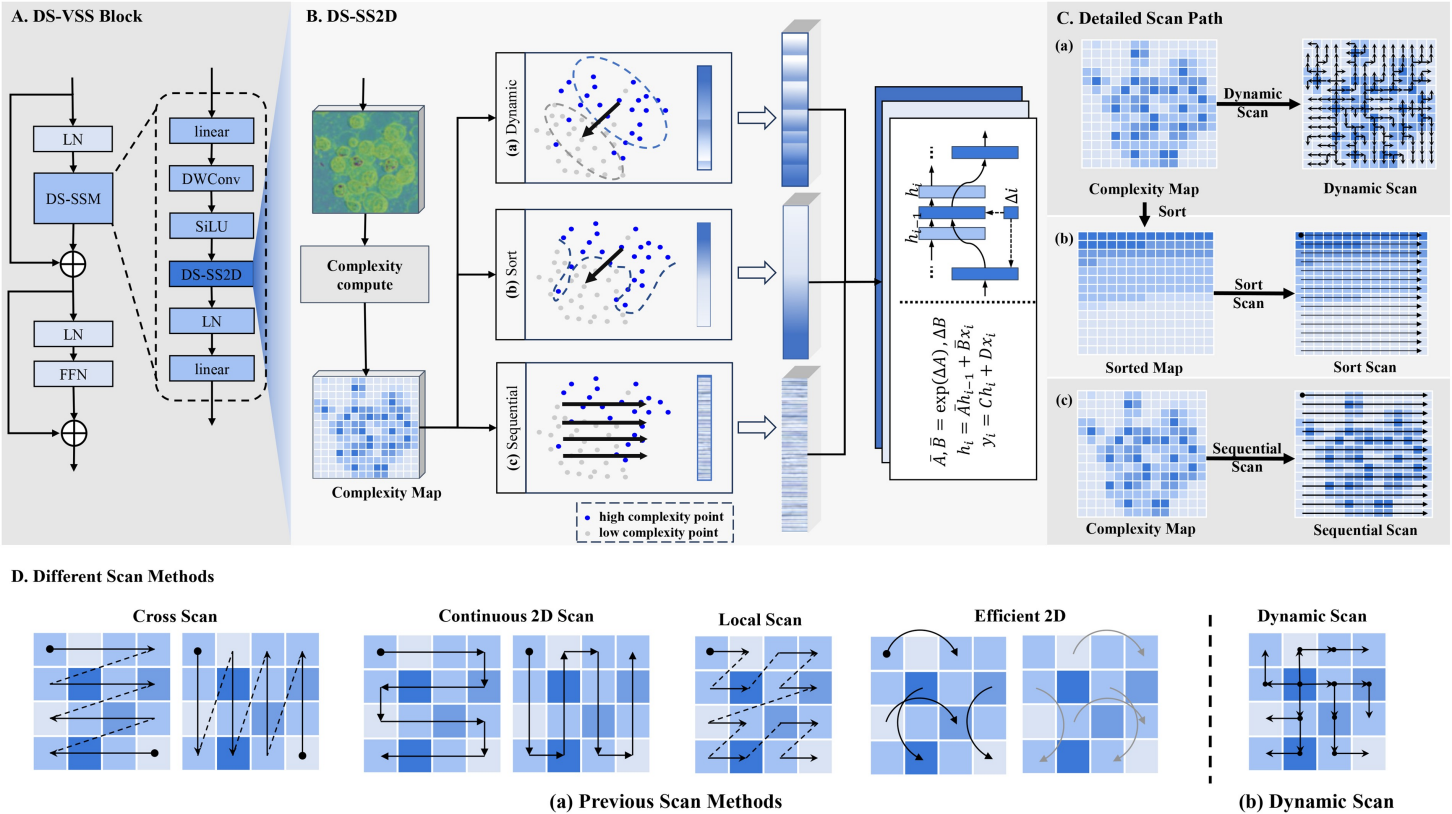
Illustration of Dynamic Scan Module workflow. (A) Shows the network architecture of the DS-VSS Block. (B) Shows the detailed process of DS-SS2D. Calculate the complexity map, then perform dynamic, sorting, and sequential scans separately, and then send to the SSM module. Three point maps show the differences between the three scanning methods. Dynamic scan follows a trend from high complexity areas to low complexity areas. Sort scan follows the absolute complexity value from high to low. Sequential scan ignores the value and focuses only on the position. (C) Shows the detailed path of B, where (a), (b), and (c) correspond to dynamic, sort, and sequential in B, respectively. (D) Shows the (a) previous scanning method and our (b) dynamic scanning path. Shades of color represent $F_{\mathrm{complex}}$ values in the complexity map.

As shown in Fig. 2 is the detail of the Dynamic Scanning SS2D(DS-SS2D) block. This module aims to enhance the target and background boundary differences and maintain the spatial continuity in the target region during the scanning process. We design a dynamic scanning method based on the intensity gradient and local feature complexity of the image, which will give priority to the regions with high intensity variation and local feature complexity.

Figure 2A illustrates the composition of the DS-VSS block. It consists of two Layer Normalization layers, a Feed-Forward Network (FFN) layer, and the core DS-SSM module. The DS-SSM includes Depthwise Separable Convolution (DWConv), the SiLU activation function, and a specially designed DS-SS2D submodule. The following will describe our DS-SS2D block in detail.

#### 2.3.1. Calculation of complexity map

As is shown in Fig. 2B, for input *I*, first obtain intensity gradient and local feature complexity, where the intensity gradient is extracted using the Sobel (2014) operator, and calculate the complexity map $F_{\mathrm{complex}}$ as follows:


(4) $$\begin{array}{c} \;G_{x}(x,y)=\left[\begin{array}{ccc} -1 & 0 & +1\\ -2 & 0 & +2\\ -1 & 0 & +1 \end{array}\right]*I(x,y), \end{array}$$

(5) $$\begin{array}{c} \;G_{y}(x,y)=\left[\begin{array}{ccc} -1 & -2 & -1\\ 0 & 0 & 0\\ +1 & +2 & +1 \end{array}\right]*I(x,y) \end{array}$$

(6) $$\begin{array}{c} G(x,y)=\sqrt{G_{x}{\left(x,y\right)}^{2}+G_{y}{\left(x,y\right)}^{2}} \end{array}$$

Where * denotes convolution operation, I(x,y) denotes original grayscale image, $G_{x}(x,y)$ denotes gradient in horizontal direction, $G_{y}(x,y)$ denotes gradient in vertical direction, and G(x,y) denotes gradient magnitude of this point.

The local feature complexity is calculated using the Local Binary Patterns (Van der Walt *et al*. 2014) formula as follows:


(7) $$\mathrm{LBP}(x,y)=\sum_{p=0}^{P-1}s(I_{p}-I(x,y))\cdot 2^{p}$$

Where $I_{p}$ denotes the *p*-th pixel value in the neighborhood, *p* denotes the number of neighborhood sampling points, s(·) is a step function to compare pixel values. Normalizing and linearly weighting the obtained features, the feature $F_{\mathrm{complex}}$ complexity formula is obtained:


(8) $$\begin{array}{c} F_{\mathrm{complex}}(x,y)=w_{1}\cdot \frac{\sqrt{G_{x}{\left(x,y\right)}^{2}+G_{y}{\left(x,y\right)}^{2}}}{\max(G)}\\ +w_{2}\cdot \frac{\sum_{p=0}^{P-1}s(I_{p}-I(x,y))\cdot 2^{p}}{\max(C)} \end{array}$$

Sort each region according to $F_{\mathrm{complex}}$ and get the final scanning order.

#### 2.3.2. Dynamic scanning path

The process of generating a dynamic scanning path is shown in Fig. 2B and C. After obtaining the complexity map, traverse the whole map based on the values of the complexity map to form a dynamic scan expansion. Next, sort all points of the complexity map to get a sorted map, and then perform sequential scanning to get the sorted scan expansion. Finally, merging the three expansions, which keeps the sequence feature values gradually decreasing and also maintains spatial continuity. Notably, although dynamic scan expansion is not an absolute sequential decline, it shows an overall downward trend. Fig. 2D shows the difference between the dynamic scan and the previous scanning methods. The specific descriptions are as follows.

Given an input feature map $X^{L}$ where *L* is the layer index of the feature map, we regard it as an undirected four-m-connected graph setting up the scanning paths. Regard the previously obtained feature complexity map $F_{\mathrm{complex}}$ as the weight matrix $G_{f}$, visit the point with the largest feature value in the neighbors of the previous point in a recursive way. In detail, We use the breadth-first search method to process the image and generate scan paths, ensuring that each scan is a localized region with the largest feature values. Thus, the spatial state model is constructed to keep feature continuity while prioritizing important features. The specific details are shown in Algorithm 1.

Algorithm 1 Dynamic Scaning

**Input:** input matrix $X_{l-1}$; feature matrix $G_{f}$

**Output:** output matrix $X_{l}$

Initialize

**Scan Path:** 

$\mathrm{Pat}h_{1},\ldots Path_{m}\leftarrow \mathrm{BFS}(G_{f}),\mathrm{Sort}(\mathrm{BFS}(G_{f}))$

$T_{l-1}^{P_{1}},\ldots ,T_{l-1}^{P_{m}}\leftarrow \mathrm{DynamicScaning}(X_{l-1},\mathrm{Pat}h_{1},\ldots ,\mathrm{Pat}h_{m})$

/* each $T_{i}^{-1}$ corresponds to the inverse mapping of Path${\;}_{i}$ */

**for** *o* in {1,…,m}  **do** 

 $T_{l-1}^{o^{\prime }}\leftarrow \text{Norm}(T_{l-1}^{o})$

 $z_{o}:\leftarrow {\text{Linear}}_{z_{o}}(T_{l-1}^{o^{\prime }})$, $x_{o}:\leftarrow {\text{Linear}}_{x_{o}}(T_{l-1}^{o^{\prime }})$

 $x_{o}^{\prime }:\leftarrow \text{SiLU}({\text{Conv}1d}_{o}(x_{o}))$

 $B_{o}:\leftarrow {\text{Linear}}_{o}^{B}(x_{o}^{\prime })$, $C_{o}:\leftarrow {\text{Linear}}_{o}^{C}(x_{o}^{\prime })$

 $\Delta_{o}:\leftarrow \;\text{log}(1+\text{exp}({\text{Linear}}_{o}^{\Delta }(x_{o}^{\prime })+{\text{Parameter}}_{o}^{\Delta }))$

 /* shape of Parameter ${\;}_{o}^{A}$ is $\left(C^{\prime },D\right)$ */

 ${\bar{A}}_{o}:,{\bar{B}}_{o}:\leftarrow \text{Disc}(\Delta_{o},{\;\text{Parameter}}_{o}^{A},B_{o})$

 $y_{o}:\leftarrow \text{SSM}({\bar{A}}_{o},{\bar{B}}_{o},C_{o})(x_{o}^{\prime })$

 $y_{o}^{\prime }:\leftarrow y_{o}⊙\text{SiLU}(z_{o})$

**end for** 

$y_{1}^{\prime },\ldots ,y_{m}^{\prime }:\leftarrow \mathrm{Restore}(y_{1}^{\prime },\ldots ,y_{m}^{\prime })$ $X_{l}:\leftarrow {\text{Linear}}^{T}(y_{\mathrm{Raw}}^{\prime }+y_{1}^{\prime },\ldots ,y_{m}^{\prime })+X_{l-1}$

**return**  $X_{l}$

### 2.4. Local aware module

To address the challenges of detail prediction and small object detection, we introduce a local aware module that performs pixel-level regional processing on images. This module is used in the initial stage of the image processing flow, designed to enhance the perception of local details.

As is shown in Fig. 1C(a), before the image is processed by patching, the input is firstly partitioned locally, and the SSM module is processed once at the pixel level. Previous SSM-based network models are usually processed after the image is patched, which ignores many fine-grained pixel-level features, and this module is built to overcome this problem. By designing the partitions to be of the target scale size, it allows each region to efficiently extract fine-grained features. Unlike CNN-based networks, though both extract fine-grained features, our approach can achieve long-distance modeling results.

In details, given an input image $I\in R^{H\times W}$, it is first divided into $n_{h}\times n_{w}$ subregions of size $\left({H/n}_{h},{W/n}_{w}\right)$. Next, each subregion is fed into an independent SSM module that does not share parameters; the relationship between locally neighboring pixels is better modeled, and detailed information can be better extracted.

## 3. Experiments and results

### 3.1. Dataset details

We evaluated the performance of DyMamba on six microscopy image segmentation datasets, including *NeurIPS-CellSeg* (https://neurips22-cellseg.grand-challenge.org/) (Ma *et al*. 2024b), *Cellpose* (https://www.cellpose.org/dataset) (Stringer *et al*. 2021), *Lucchi* (https://www.epfl.ch/labs/cvlab/data/data-em/) (Lucchi *et al*. 2012), *LiveCell* (https://sartorius-research.github.io/LIVECell/) (Edlund *et al*. 2021), *MitoEM-R* (https://mitoem.grand-challenge.org/) (Wei *et al*. 2020), and *Fluo-C2DL-MSC* (https://celltrackingchallenge.net/2d-datasets/) (Maška *et al*. 2014). These datasets were chosen among various resolutions and image modalities. The raw image is shown in Fig. 4.

For brevity, we refer to NeurIPS-CellSeg as CellSeg and Fluo-C2DL-MSC as MSC in the following.

**Figure 3:**
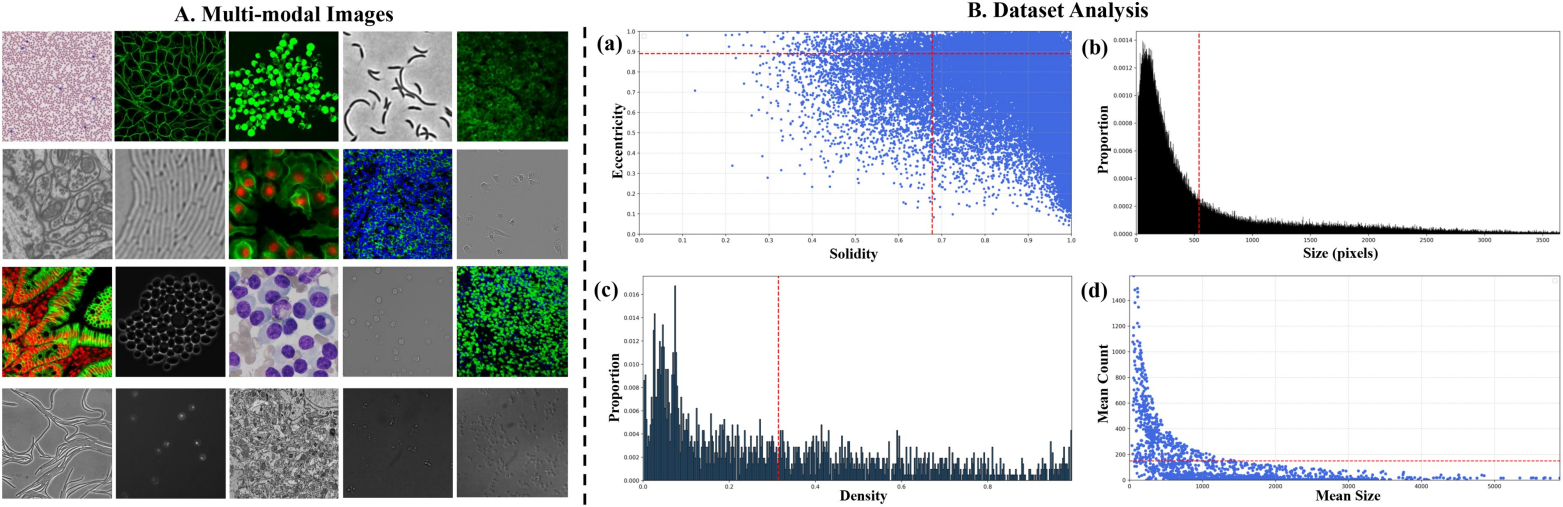
Overview and analysis of the datasets. Figure (A) Shows microscopy images of multiple modalities. Figure (B) shows the morphology and distribution statistics of the target. (a) Solidity-Eccentricity Scatter plot: Each point corresponds to a segmentation target, with Solidity on the horizontal axis and Eccentricity on the vertical axis; (b) Target Size distribution histogram: The horizontal axis represents the area of a single target(pixels²); (c) Target density distribution histogram: The horizontal axis represents the target distribution density in each image; (d) Image-level scatter plot: the *x*-axis represents the mean object size per image, and the *y*-axis represents the mean number of objects per unit area (5,12,512). The red dotted lines correspond to the mean value of each measure.

Figure 3 presents an overview and statistics of the dataset. The multimodality of microscopic images primarily stems from the differences in imaging techniques (e.g. bright field, fluorescence, phase contrast, differential interference contrast) and the types of inspected tissues (Lee *et al.* 2022). In addition, the shape and size of cells themselves vary with type, period and magnification, leading to instance-level heterogeneity. At the annotation level, different annotators have varying standards for boundary selection, contour, recognition and segmentation, which introduces label noise and reduces model performance. In conclusion, factors such as technology, tissue, cells and imaging quality jointly constitute the complex multimodal distribution of microscopic images, which need to be systematically considered in algorithm design.

**Figure 4:**
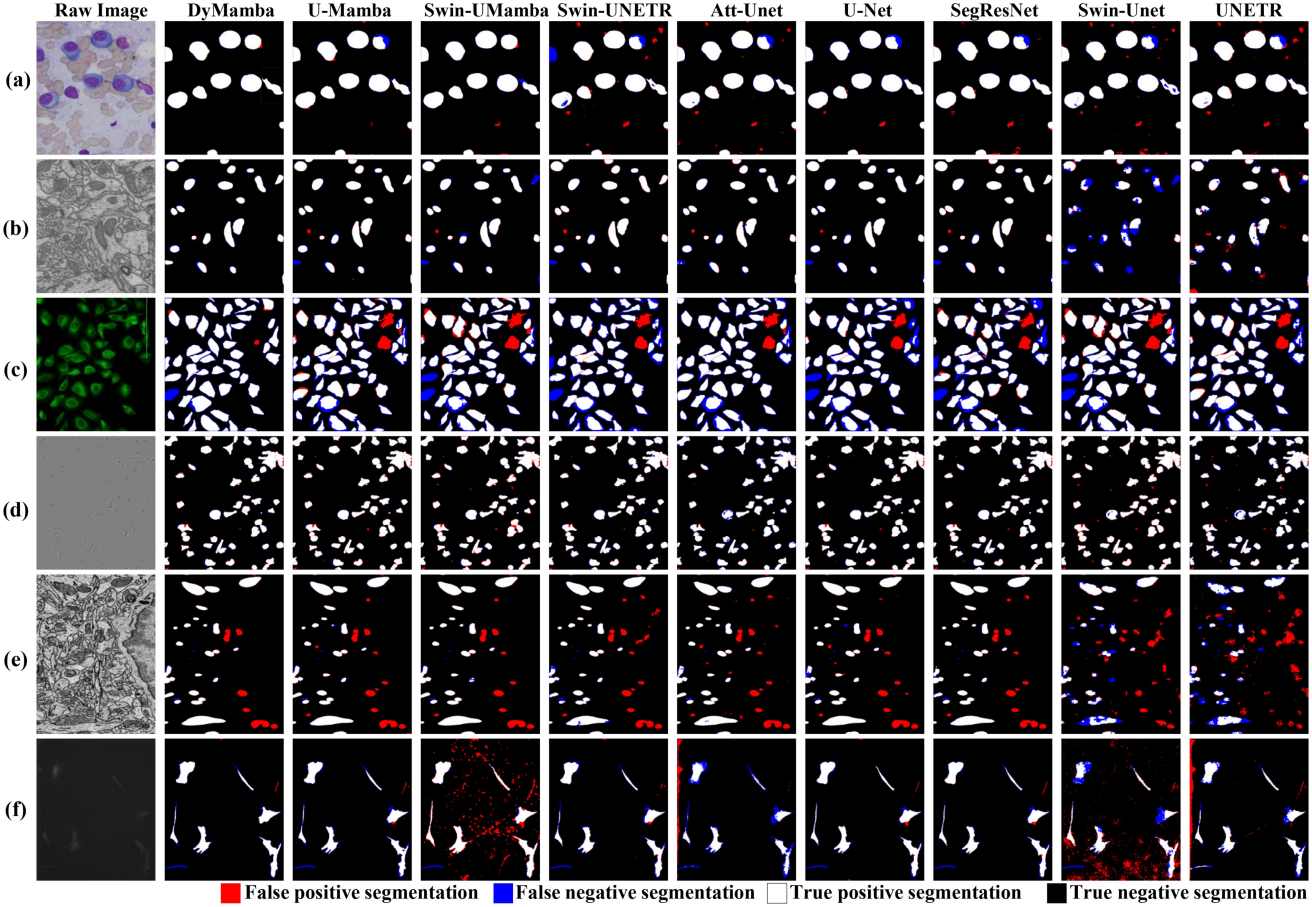
Qualitative results of different methods. Figure (a–f) correspond to the six public datasets: CellSeg, Lucchi, Cellpose, LiveCell, MitoEM-R, and Fluo-C2DL-MSC. To highlight subtle differences, some images have been cropped and enlarged. Each subfigure displays, from left to right: the original image and segmentation results from all comparison methods. The overall arrangement follows a progression from poorer to superior segmentation performance, facilitating intuitive comparison. In the segmentation result image, red indicates false positive segmentation, blue indicates false negative segmentation, white indicates true positive segmentation, and black indicates true negative segmentation. Our Dymamba method demonstrates high consistency with ground truth across various datasets, significantly outperforming other methods.

### 3.2. Implemetation details

#### 3.2.1. Training details

Our model is implemented in PyTorch 2.0.1-cuda11.8, where we substitute the AdamW optimizer and train 300 epochs from scratch with a warm-up strategy of 20 epochs. The code is implemented on top of U-Net (Ronneberger *et al*. 2015). During training, the learning rate is decayed using a cosine scheduler. All experiments were performed on a computing platform with four NVIDIA A100 GPU (40GB), but no multi-GPU training.

During training, we randomly sampled 512 × 512 image patches. During inference, we used a sliding window to crop 512 × 512 image patches with a stride of 384. Data augmentation was not used during testing.

#### 3.2.2. Loss function

We employ a combined approach of cross-entropy loss and Dice loss in selecting the loss function. This approach balances pixel-level classification accuracy with small-object region overlap, thereby mitigating class imbalance and enhancing edge segmentation precision. The loss function can be formulated as follows:


(9) $$L_{\mathrm{Seg}}=L_{\mathrm{Dice}}+L_{CE}$$

#### 3.2.3. Data augmentation

We provide the combined augmentation policy in Table 1.

**Table 1:** Augmentation strategies.

Strategy	Implementation details
SpatialPadd	Pad the image to (5,12,512)
RandSpatialCropd	Crop the image to (5,12,512) if oversized
RandAxisFlipd	Flip the image along an axis, prob = 0.5
RandRotate90d	Rotate the image and label by 90 degrees along the specified axis, prob = 0.5, spatial_axes = [0, 1]
RandGaussianNoised	Add Gaussian noise to the image, prob = 0.25, mean = 0, std = 0.1
RandAdjustContrastd	Adjust the image contrast, prob = 0.25, gamma = (1, 2)
RandGaussianSmoothd	Apply Gaussian smoothing to the image, prob = 0.25, sigma_x = (1, 2)
RandHistogramShiftd	Perform histogram shift on the image, prob = 0.25, num_control_points = 3
RandZoomd	Randomly scale the image and label, prob = 0.15, min_zoom = 0.8, max_zoom = 1.5

### 3.3. Hyper-parameters

In the complexity calculation step of the DS-SS2D module, *w*1 and *w*2 are set to 0.5 and 0.5, respectively, and *p* is set to 8. The configuration of $w_{1}$ and $w_{2}$ addresses the fundamental requirements of the dynamic scanning task. Edge localization information ($w_{1}$) guides the model to capture cell boundaries, while texture discrimination information ($w_{2}$) ensures correct identification of region types. This configuration balances precise boundary localization and accurate region classification, based on the complementary symmetry and scale consistency of the information.

### 3.3. Segmentation performance

We chose three types of methods for comprehensive evaluation, including CNN-based U-Net (Ronneberger *et al*. 2015), SegResNet (Myronenko 2019), Attention-Unet (Zhu *et al*. 2022), Transformer-based UNETR (Hatamizadeh *et al*. 2022), Swin-Unet (Cao *et al*. 2022), Swin-UNETR (Hatamizadeh *et al*. 2021) and Mamba-based segmentation networks U-Mamba (Ma *et al*. 2024a), Swin-UMamba (Liu *et al*. 2024a). We used mIoU and mDice to evaluate model performance. In addition, we calculated the number of parameters in the model to estimate the size of each model. In addition, we calculated the number of parameters(#param) for each model.

Table 2 shows the segmentation performance of these segmentation datasets (CellSeg, Cellpose, Lucchi, MitoEM-R, LiveCell, and MSC). DyMamba outperforms all baseline methods, including CNN-based, transformer-based and Mamba-based networks, achieving an average improvement of 6.9% in mDice and 4.3% in mIoU over state-of-the-art methods across all datasets. Compared to the best baseline, DyMamba achieved new best performance with only 21 M parameters. On the CellSeg, Cellpose, Lucchi, MitoEM-R, LiveCell, and MSC datasets, DyMamba achieves mIoU scores of 0.819, 0.802, 0.907, 0.838, 0.869, and 0.760, respectively, outperforming all CNN, Transformer, and Mamba baselines. Additionally, despite other models achieving good results on the Lucchi dataset, DyMamba still achieved a 4.2% improvement in mIoU.

**Table 2:** Segmentation results on CellSeg, Cellpose, Lucchi, MitoEM-R, LiveCell, MSC.

Dataset		**CellSeg**		**cellpose**		**Lucchi**		**MitoEM-R**		**LiveCell**		**MSC**	
Method	params	mIoU	mDice	mIoU	mDice	mIoU	mDice	mIoU	mDice	mIoU	mDice	mIoU	mDice
*CNN-based*													
U-Net	31M.	0.656	0.791	0.737	0.839	0.799	0.888	0.739	0.828	0.799	0.888	0.730	0.845
		±0.034	±0.046	±0.117	±0.033	±0.022	±0.049	±0.034	±0.056	±0.023	±0.038	±0.037	±0.024
SegResNet	6M.	0.596	0.746	0.695	0.820	0.763	0.868	0.759	0.864	0.777	0.877	0.576	0.729
		±0.075	±0.023	±0.058	±0.025	±0.063	±0.023	±0.066	±0.054	±0.017	±0.015	±0.071	±0.057
Att-Unet	8M.	0.662	0.795	0.537	0.699	0.676	0.806	0.638	0.777	0.730	0.845	0.715	0.835
		±0.026	±0.035	±0.078	±0.026	±0.038	±0.046	±0.037	±0.025	±0.065	±0.022	±0.051	±0.033
*Transformer-based*													
UNETR	87M.	0.727	0.842	0.565	0.722	0.839	0.910	0.672	0.805	0.764	0.867	0.586	0.737
		±0.024	±0.072	±0.087	±0.052	±0.025	±0.040	±0.052	±0.045	±0.044	±0.061	±0.092	±0.078
Swin-Unet	27M.	0.678	0.807	0.541	0.703	0.690	0.819	0.604	0.717	0.785	0.884	0.523	0.688
		±0.039	±0.060	±0.005	±0.019	±0.038	±0.024	±0.046	±0.039	±0.032	±0.015	±0.071	±0.061
Swin-UNETR	22M.	0.726	0.841	0.644	0.786	0.882	0.937	0.655	0.793	0.817	0.898	0.730	0.843
		±0.040	±0.051	±0.046	±0.032	±0.025	±0.034	±0.056	±0.024	±0.058	±0.056	±0.047	±0.032
*Mamba-based*													
U-Mamba	76M.	0.760	0.864	0.616	0.762	0.866	0.928	0.748	0.854	0.796	0.887	0.623	0.768
		±0.039	±0.061	±0.067	±0.058	±0.039	±0.029	±0.061	±0.035	±0.040	±0.053	±0.060	±0.045
Swin-UMamba	60M.	0.752	0.859	0.702	0.824	0.865	0.927	0.754	0.858	0.819	0.901	0.687	0.813
		±0.017	±0.052	±0.025	±0.078	±0.027	±0.022	±0.045	±0.036	±0.053	±0.042	±0.092	±0.072
DyMamba	21M.	**0.819**	**0.901**	**0.802**	**0.883**	**0.907**	**0.951**	**0.838**	**0.912**	**0.869**	**0.930**	**0.760**	**0.863**
		±0.036	±0.024	±0.049	±0.038	±0.029	±0.040	±0.052	±0.054	±0.053	±0.030	±0.065	±0.055

Values in bold indicate the best performance.

In addition, DyMamba also has a significant advantage in terms of parameter count. Compared to UNETR with 87M parameters and U-Mamba with 76M parameters, DyMamba achieves higher performance with only 21M parameters, demonstrating that DyMamba is more efficient in terms of model complexity and computational resources. While maintaining a low parameter count, DyMamba can effectively improve segmentation accuracy.

As shown in Fig. 4, we demonstrate qualitative segmentation results of DyMamba on six datasets. These datasets are CellSeg, Lucci, Cellpose, LiveCell, MitoEM-R, and MSC, corresponding to parts (a), (b), (c), (d), (e), and (f) in the figure. We use different colors to distinguish the segmentation results: red represents false positive segmentation, blue represents false negative segmentation, white represents true positive segmentation, and black represents true negative segmentation. As shown in Fig. 4, the DyMamba method demonstrates outstanding performance across multiple datasets. In particular, on the CellSeg and Cellpose datasets, DyMamba excels in reducing false positives and false negatives in segmentation, indicating its advantages in terms of accuracy and recall. Additionally, on the Lucci and LiveCell datasets, DyMamba also exhibits high segmentation accuracy, with its segmentation results being more precise compared to other state-of-the-art methods.

Furthermore, It should be noted that the Kvasir dataset is not a dataset for microscopic cell segmentation tasks. We selected this dataset to verify the generalization ability of our model. As shown in Table 2, our model still achieved excellent performance on this dataset.

### 3.4. Comparison of scanning strategy

In this section, we perform experiments with different scanning strategies on the CellSeg and Cellpose datasets. These two datasets contain almost all modalities of the six selected datasets. As is shown in Fig. 2B, we choose four representative scanning methods for comparison: Cross-Scan (Liu *et al*. 2024b), Continuous 2D Scan (Yang *et al*. 2024), Local Scan (Huang *et al*. 2024), Efficient 2D Scan (Pei *et al*. 2025). The results in shown in Table 3.

**Table 3:** Results of different scanning strategies on the CellSeg and Cellpose dataset.

Dataset	Method	mIoU	mean Dice
cellseg	Cross Scan	0.785 ± 0.040	0.878 ± 0.048
	Continuous 2D	0.759 ± 0.016	0.864 ± 0.033
	Local Scan	0.774 ± 0.089	0.871 ± 0.058
	Efficient 2D	0.742 ± 0.070	0.852 ± 0.011
	Random Scan	0.718 ± 0.067	0.837 ± 0.012
	Dynamic Scan	**0.819 ± 0.036**	**0.901 ± 0.024**
cellpose	Cross Scan	0.745 ± 0.053	0.826 ± 0.066
	Continuous 2D	0.727 ± 0.064	0.806 ± 0.024
	Local Scan	0.718 ± 0.046	0.821 ± 0.049
	Efficient 2D	0.722 ± 0.037	0.832 ± 0.062
	Random Scan	0.693 ± 0.051	0.761 ± 0.029
	Dynamic Scan	**0.802 ± 0.049**	**0.883 ± 0.038**

Values in bold indicate the best performance.

**Figure 5:**
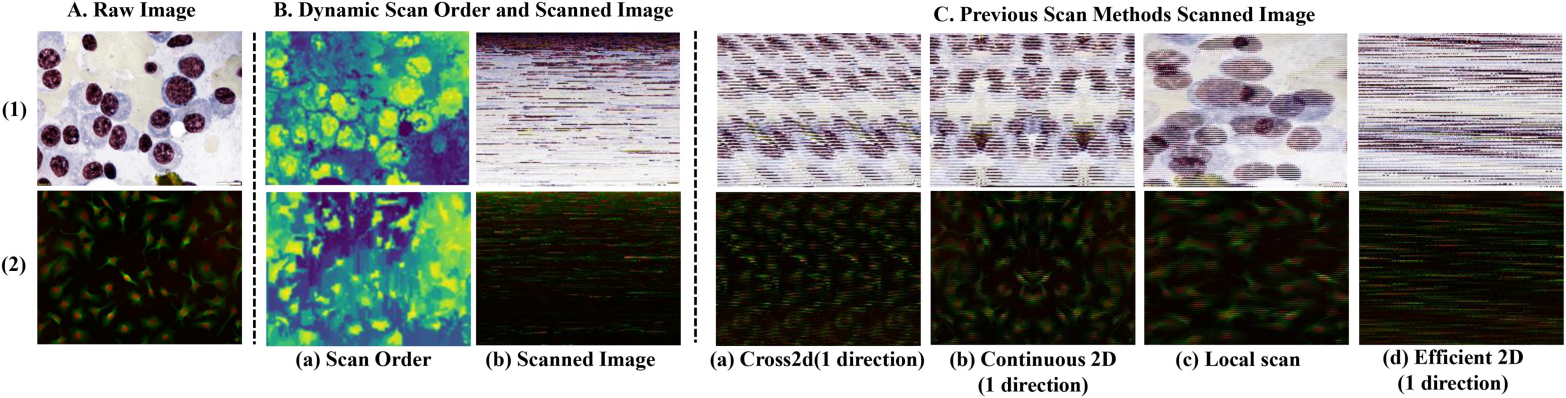
Dynamic Scan Visualization. (A) Raw Image. (1) Brightfield microscopy image from CellSeg dataset, (2) fluorescence microscopy image from Cellpose dataset. (B) Dynamic Scan Order and Scanned Image. (a) Scan order: Higher brightness indicates earlier scanning, with visible target areas scanned first. (b) Scanned image: Following the scan order in (a), target pixels are concentrated in the front region (C). Previous Scan Methods Scanned Image. The four previous scanning methods don’t scan target areas with priority, and also introduce spatial discontinuities. For methods that scan from multiple directions and obtain similar results, we select one representative direction for demonstration.

As shown in Fig. 5, we select two images (brightfield microscopy image and fluorescence microscopy image) from the CellSeg and Cellpose datasets. Fig. 5B demonstrates that the dynamic scanning method prioritizes scanning the target area. Fig. 5B(a) illustrates the scan order, where brighter areas indicate earlier scanning. It is intuitively evident that the target region requiring segmentation was scanned first, while maintaining the spatial continuity of these target regions. Fig. 5B(b) shows the result of scanning and arranging each pixel from bright to dark in the order specified in Fig. 5B(a), revealing that the target region is positioned at the front. Fig. 5C displays scanned images from previous methods, where the target region is spread uniformly without prioritizing its scanning and introducing spatial discontinuities. For methods that scan from multiple directions and obtain similar results, we select one representative direction for demonstration.

### 3.5. Parameter research

#### 3.5.1. Study on the number of layers

**Figure 6:**
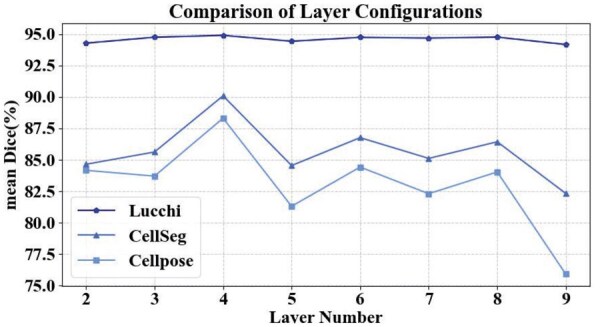
Comparison of Layer Configurations. The mDice score for all datasets reached its maximum at 4 layers. As the number of layers increased, the performance of CellSeg and Cellpose slightly decreased, while Lucchi’s performance remained relatively stable.

For the Settings of network parameters L1, L2, L3, and L4, the previous methods basically set them to 2,2,9,2 (Zhu *et al*. 2024, Liu *et al*. 2024b). To ensure the segmentation effect while saving space and time, we conduct an experiment on the setting of parameter L3. The experiment is implemented on Lucchi, Cellpose and CellSeg datasets. The experimental setup and effects are shown in Fig. 6 and Table 4. The experimental results show that as the value of L3 increases, the effect first rises and then falls, while the number of parameters and Flops both rise. Finally, we set L3 to 4.

**Table 4:** Comparison of layer configurations on the Lucchi, CellSeg and cellpose dataset.

Dataset			Lucchi	CellSeg	Cellpose
Layer	GFLOPs	#Params	mDice	mDice	mDice
2	21.93	18.99M	0.943	0.847	0.842
3	23.20	20.23M	0.948	0.856	0.837
4	24.48	21.48M	**0.949**	**0.901**	**0.883**
5	25.76	22.72M	0.944	0.846	0.813
6	27.03	23.97M	0.943	0.868	0.844
7	28.31	25.21M	0.947	0.851	0.823
8	29.59	26.46M	0.948	0.864	0.840
9	30.86	27.70M	0.942	0.823	0.759

Values in bold indicate the best performance.

#### 3.5.2. Study on the patch number of local aware module

**Figure 7:**
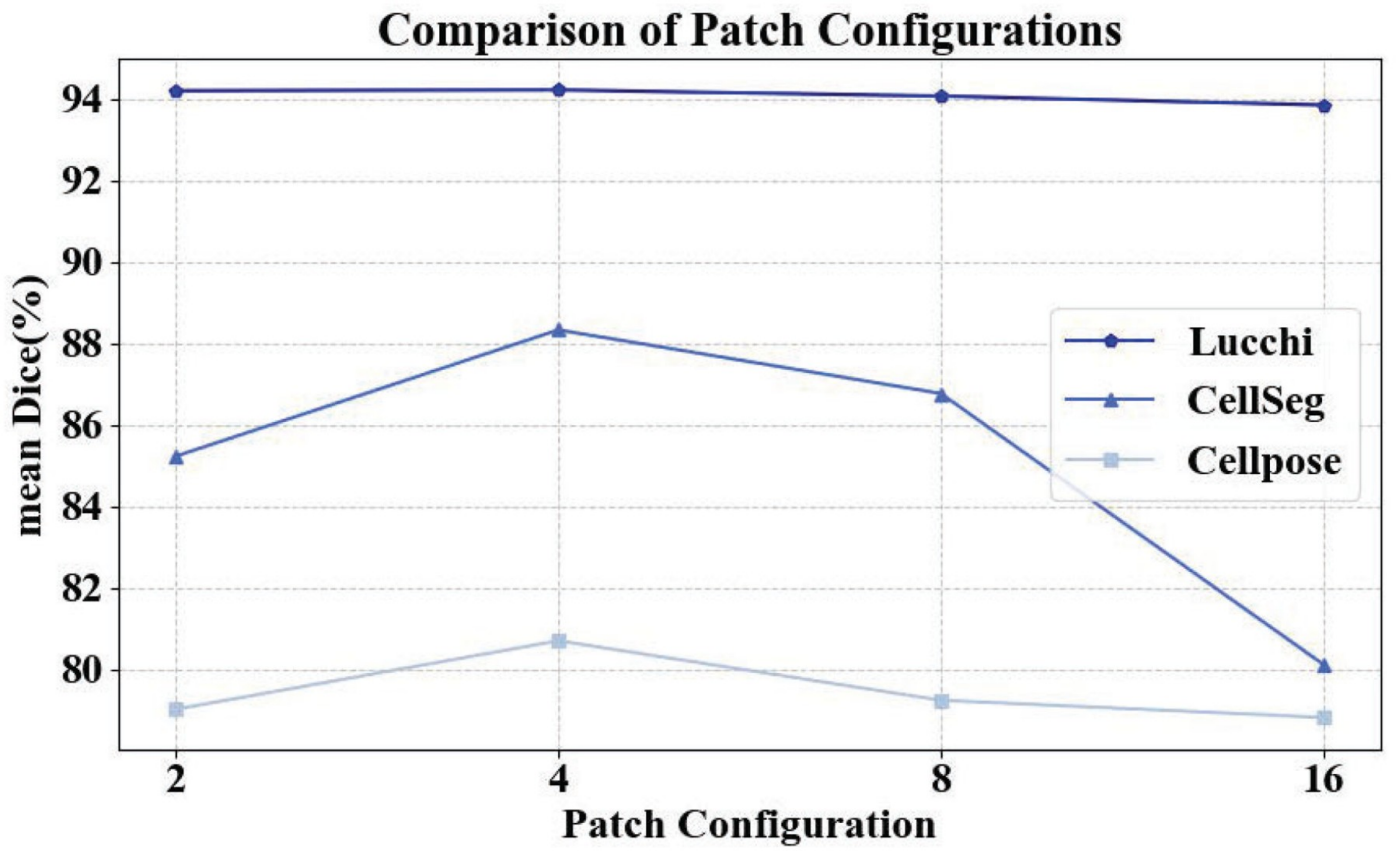
Comparison of Patch Configurations. The Lucchi dataset maintains an mDice coefficient of approximately 94% across all configurations, indicating that patch num has a relatively minor impact on the Lucchi dataset. The CellSeg, MitoEM-R, and Cellpose datasets achieve peak performance when patch num is set to 4, followed by a subsequent decline.

To explore the influence of patch size on the local aware module, we set the patch size to 2,4,8 and 16. The experiment is implemented on Lucchi, Cellpose and CellSeg datasets, and the segmentation results are shown in Fig. 7. Based on the results in the figure, it can be seen that the patch number set to 4 works best. As can be seen from the Fig. 7, the Lucchi dataset is insensitive to changes in the number of patches. The target size and spatial distribution in the Lucchi dataset are relatively uniform, so the potential gain from adaptive scanning strategies on this dataset is inherently limited, which precisely validates the rationality of our method design.

### 3.6. Ablation study & qualitative results

We perform ablation experiments on CellSeg, Cellpose, and MitoEM-R datasets to quantitatively evaluate the specific contributions of each key module in Dy-Mamba to overall performance. Given that the Lucchi dataset has consistent object sizes, clear boundaries, and a moderate amount of data, all models performed very well. Therefore, we select MitoEM-R as the dataset for scanning electron microscopy mode in the ablation experiments. Results are summarized in Table 5. The experimental setup is as follows: using a network with all improved modules removed as the unified baseline, we sequentially added the dynamic scan (DS) module and the local aware (LA) module. After introducing only the DS module, the model achieved an average mIoU improvement of 3.3% across all three datasets, demonstrating that long-range dependency modeling via dynamic scanning significantly enhances global modeling capabilities. When the LA module was added while retaining the DS module, the overall mIoU further improved by 2.4%. This indicates that supplementary information from local textures and edge details effectively compensates for fine-grained features potentially lost during long-range modeling. Comparative experiments reveal that the standalone contribution of the DS module (↑3.3%) exceeds that of the LA module (↑2.1%), indicating the dynamic scanning strategy holds greater advantage in balancing long-range dependencies and spatial continuity. When combined, their performance gains are not simply additive, validating the necessity of global-local coupled modeling.

**Table 5:** Ablation study on the CellSeg, Cellpose and MitoEM-R dataset.

Dataset	Settings	mIoU	mean Dice
Cellseg	Backbone	0.782 ± 0.025	0.878 ± 0.036
	w/DS	0.812 ± 0.028	0.896 ± 0.045
	w/LA	0.791 ± 0.017	0.883 ± 0.063
	w/DS, LA	**0.819 ± 0.034**	**0.901 ± 0.022**
Cellpose	Backbone	0.737 ± 0.020	0.848 ± 0.044
	w/DS	0.762 ± 0.022	0.865 ± 0.058
	w/LA	0.752 ± 0.069	0.858 ± 0.037
	w/DS, LA	**0.792 ± 0.016**	**0.883 ± 0.034**
MitoEM-R	Backbone	0.759 ± 0.025	0.864 ± 0.036
	w/DS	0.804 ± 0.028	0.893 ± 0.045
	w/LA	0.797 ± 0.017	0.888 ± 0.063
	w/DS, LA	**0.838 ± 0.034**	**0.912 ± 0.022**

Backbone: DyMamba network removes the local aware module and dynamic scanning algorithms.

“w/DS”: only add the dynamic Scanning module on the backbone. “w/LA”: only add the local aware module on the backbone. “w/DS, LA”: add dynamic scanning and local aware modules to the backbone.

Values in bold indicate the best performance.

**Figure 8:**
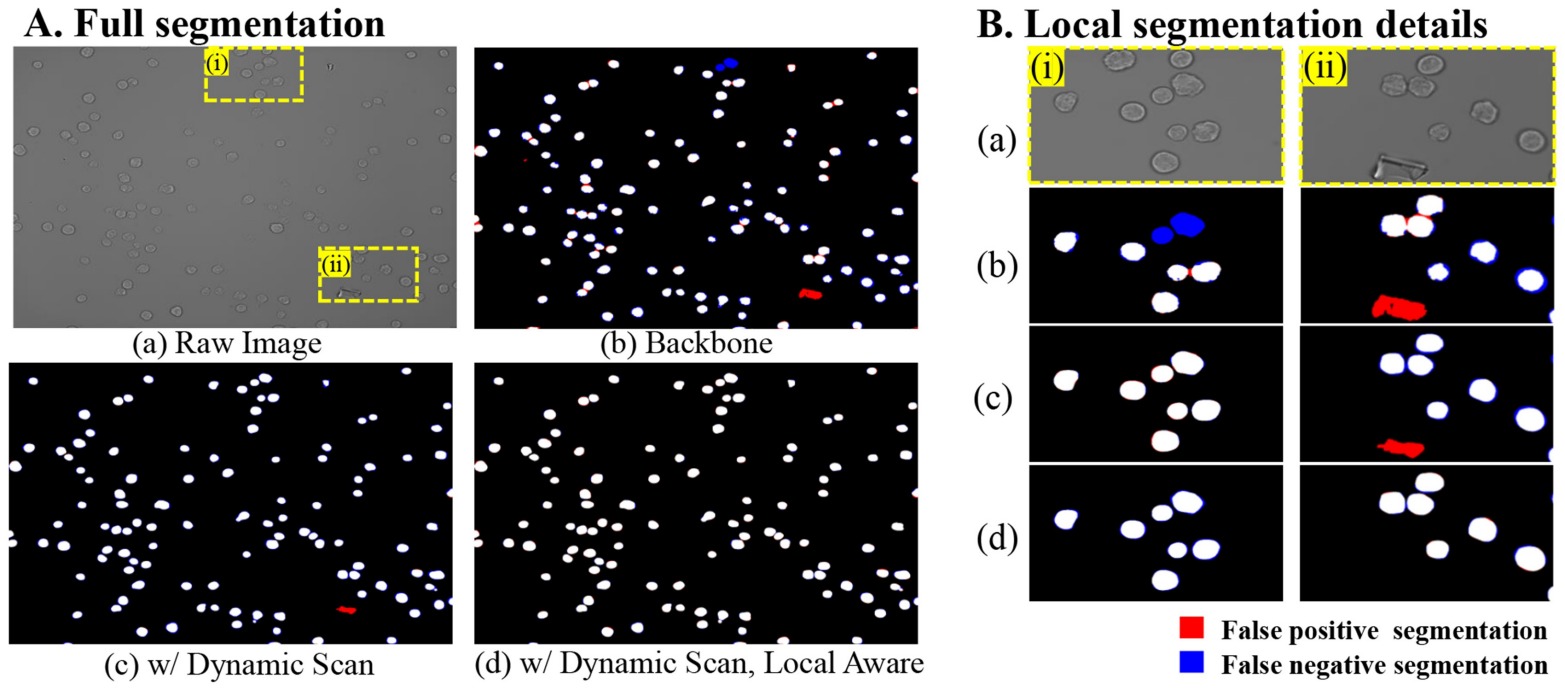
Visualization of Ablation Studies on the CellSeg Dataset. (A) Comparison of ablation experiments across the full image. (B) Magnified comparison of the yellow dashed-line region in A: (a–d) raw image, baseline, with dynamic scan module, and with dynamic scan module and local-aware module. (i), (ii): further magnify the dashed-line regions in A, highlighting areas not predicted by the baseline and local edge sharpness.

Figure 8 presents visualization results on the CellSeg test set: Adding the DS module alone (Fig. 8B(c)) successfully recalls two cells missed by the baseline. Introducing the LA module subsequently (Fig. 8B(d)) markedly reduces edge artifacts in the recalled cells. This further demonstrates that the DS module and LA module complement each other in long-range modeling and local detail refinement, respectively, resulting in more precise and smoother segmentation.

## 4. Conclusion

The study aims to introduce a new framework, DyMamba, for microscopy image segmentation. Our central idea is to eliminate spatial discontinuities as much as possible while maintaining long-range modeling. Specifically, the dynamic scan module enables dynamic scan path settings, and the local aware module improves local feature modeling capability. Experiments on six datasets show that our DyMamba outperforms existing algorithms for microscopy image segmentation under various imaging conditions and multiple modalities.
